# Clinical utility of methicillin-resistant *Staphylococcus aureus* nasal polymerase chain reaction (PCR) assays beyond respiratory infections

**DOI:** 10.1017/ash.2022.256

**Published:** 2022-06-30

**Authors:** Haley M. Noeldner, Zachary J. Bliek, Nephi E. Jones, Ryan D. Prusa, Melissa S. Wilkinson, Amanda M. Bushman, Rossana Rosa

**Affiliations:** 1 Department of Pharmacy, UnityPoint Health–Des Moines, Des Moines, Iowa; 2 Podiatry Service, Department of Surgery, Iowa Methodist Medical Center, Des Moines, Iowa; 3 Department of Medicine, University of Iowa–Des Moines Campus, Des Moines, Iowa; 4 Infectious Diseases Service, UnityPoint Health–Des Moines, Des Moines, Iowa (Present affiliations: Department of Pharmacy, Riverwood Healthcare Center, Aitkin, Mnnesota [H.M.N.] and Department of Quality and Patient Safety, Jackson Health System, Miami, Florida [R.R.])

## Abstract

We estimated the predictive value of methicillin-resistant *Staphylococcus aureus* (MRSA) nasal polymerase chain reaction (PCR) for blood, bone, and soft-tissue cultures. The specificities were 88.8%, 88.5%, and 92.7% for all cultures, blood cultures, and bone and soft-tissue cultures respectively, and the negative predictive values were 99.3%, 99.8%, and 92.7% respectively.

The use of methicillin-resistant *Staphylococcus aureus* (MRSA) nasal polymerase chain reaction (PCR) has been shown to be an efficient way to detect the presence of MRSA colonization compared to traditional culture methods.^1^ Strong data support the use of MRSA nasal PCR to predict the absence of MRSA in pneumonia and guide therapeutic decisions.^2,3^ Studies evaluating the predictive value of MRSA nasal PCR on nonlung infections are fewer, but the body of evidence on this subject is expanding.^4–6^ We sought to determine the clinical utility of MRSA nasal PCR assays beyond respiratory indications by estimating its predictive value for clinical cultures from blood, bone, and soft-tissue infections.

## Methods

This retrospective cohort study was performed across 3 hospitals that are part of an integrated health system in Des Moines, Iowa. During the study period, the local antibiogram showed that 40% of *Staphylococcus aureus* isolates tested were methicillin resistant. The study period was March 1, 2019, to February 29, 2020. We included patients aged ≥18 years who had a MRSA nasal PCR (GeneXpert GX-XVI and DX System version 4.8 software, Cepheid, Sunnyvale, CA) during a hospital admission and had a clinical culture obtained within 3 days of the MRSA PCR order date. Clinical cultures included blood, soft-tissue, deep podiatric wound (indicating a podiatrist was both the ordering provider and physically collected the specimen), bone, joint aspirate, or synovial fluid cultures. Clinical samples were processed following standard procedures throughout the study period; selective media for staphylococci was not used. Only 1 encounter per patient was analyzed, but some patients contributed >1 type of specimen (ie, both blood and tissue). If an individual had multiple hospital encounters within the study period, only the first encounter in which both MRSA nasal PCR and clinical culture data were available was evaluated. At the facilities included, a MRSA nasal PCR can be ordered by any clinician (irrespective of patient diagnosis), clinical pharmacist (in patients with a vancomycin order for suspected pneumonia) or nurse in the intensive care unit (as part of routine admission screening). The test is part of an electronic order set for pneumonia and sepsis, but it is not automatically obtained. Baseline data including age, sex, and risk factors for MRSA infections (ie, history of diabetes mellitus and/or receiving any form of dialysis prior to hospital admission) were collected via manual review of the electronic medical record (Epic, Verona, WI). This study protocol was reviewed and approved by the UnityPoint Health–Des Moines Institutional Review Board (IRB).

### Statistical analysis

Descriptive statistics using medians and proportions were used to analyze the data. Differences in proportions of patients with growth of MRSA in a clinical culture and MRSA nasal PCR status were calculated using the χ^2^ test. Using growth in culture as the gold standard, the sensitivity, specificity, positive predictive value (PPV), and negative predictive value (NPV) of MRSA nasal PCR were estimated. Analysis was conducted collectively for the total cohort and then separately for blood culture and bone and soft-tissue samples.

## Results

In total, 1,989 patients were included in data analysis. 54.6% of the patient population was male. The median age was 66 years (interquartile range, 54–77). At baseline, 659 patients (33.1%) had a diagnosis of diabetes mellitus, and 75 patients (3.8%) were on renal replacement therapy.

Blood cultures were obtained on 1,953 (98.2%) of 1,989 patients, and 171 patients (8.6%) had a bone or soft-tissue culture obtained. A positive MRSA PCR was detected in 245 patients (12.3%) (Table 1). Growth of MRSA was noted in 22 (1.1%) of 1,953 blood cultures, and in 20 (11.7%) of 171 bone or soft-tissue cultures (Table 1). The sensitivities of MRSA PCR for determining presence of MRSA in culture were 67.5% for any clinical culture, 81.8% for blood cultures, and 55.0% for bone or soft-tissue cultures, whereas the specificities were 88.8% for all cultures, 88.5% for blood cultures, and 92.7% for bone or soft-tissue cultures (Table 2). The positive predictive values (PPVs) were 11.0% for all cultures, 7.5% for blood cultures, and 50.0% for bone and soft-tissue cultures. The negative predictive values (NPVs) were 99.3% for all cultures, 99.8% for blood cultures, and 92.7% for bone and soft-tissue cultures (Table 2).

**Table 1.** Results of MRSA Nasal PCR Compared to Clinical Cultures

Nasal PCR	Any Clinical Culture			Blood Cultures			Bone and Soft-Tissue Cultures^a^		
	MRSA Positive	MRSA Negative	Total	MRSA Positive	MRSA Negative	Total	MRSA Positive	MRSA Negative	Total
MRSA positive	27	218	245	18	223	241	11	11	22
MRSA negative	13	1,731	1,744	4	1,708	1712	9	140	149
Total	40	1,949	1,989^b^	22	1,931	1,953^b^	20	151	171^b^

Note. MRSA, methicillin-resistant *Staphylococcus aureus*; PCR, polymerase chain reaction.

a Includes deep podiatric wound, bone, joint aspirate, or synovial fluid cultures.

b The total number of patients with clinical cultures is lower than the sum of blood plus bone/soft tissue cultures since some patients had both types of samples collected.

**Table 2.** Prevalence, Sensitivity, Specificity, Positive Predictive Value (PPV), and Negative Predictive Value (NPV) of MRSA Nasal PCR for Blood or Bone Soft-Tissue Cultures

Variable	Any Clinical Culture		Blood Cultures		Bone and Soft-Tissue Cultures^a^	
	%	95% CI	%	95% CI	%	95% CI
Prevalence	12.3	10.9–13.8	12.3	10.9–13.9	12.9	8.2–18.8
Sensitivity	67.5	50.9–81.4	81.8	59.7–94.8	55.0	31.5–76.9
Specificity	88.8	87.3–90.2	88.5	86.9–89.8	92.7	87.3–96.3
PPV	11.0	7.4–15.6	7.5	4.5–11.6	50.0	28.2–71.8
NPV	99.3	98.7–99.6	99.8	99.4–99.9	92.7	87.3–96.3

Note. MRSA, methicillin-resistant *Staphylococcus aureus*; PCR, polymerase chain reaction; CI, confidence interval.

a Deep podiatric wound, bone, joint aspirate, or synovial fluid cultures.

## Discussion

The results of MRSA nasal PCR had a high specificity and negative predictive value for growth of MRSA in blood and bone or soft-tissue cultures. These findings contribute to a growing body of evidence on the utility of MRSA nasal PCR assays for ruling out MRSA in infections other than pneumonia.

Our results are concordant with recent studies. Petry et al^4^ reported on a cohort of 337 patients who had MRSA nasal screening via PCR and wound or tissue cultures and found specificities of 86.2% and 88.8%, respectively, with NPVs of 93.6% and 93.5%. In a large retrospective study that included >500,000 clinical cultures, with 73.7% of patients screened by PCR, Mergenhagen et al^5^ reported an overall specificity of 81.2% and NPV of 96.5% for MRSA nasal screening for any clinical culture; a specificity of 81.9% and NPV of 96.5% for blood cultures; and a specificity of 85.4% and NPV of 93.5% for sterile wounds.

The utility of incorporating results of MRSA nasal screening into therapeutic and stewardship decision for the management of pneumonia has been well established.^3^ Therefore, our results in conjunction with those of similar studies could be used to develop protocols to assess the clinical impact and safety of utilizing MRSA nasal PCR to guide decisions around anti-MRSA therapies in infections other than pneumonia.

Our study had several limitations. First, this was a retrospective cohort study, and although it included nearly 2,000 patients, a much smaller cohort of patients within the study had a bone or soft-tissue culture obtained than those who had a blood culture obtained. Although the NPV for bone or soft-tissue cultures was still >90%, the result was lower than the NPV for blood cultures, likely secondary to the smaller patient sample. Furthermore, we present data on microbiological findings only. However, based on the type of sample and collection characteristics they are presumed to represent true clinical infections. Also, there some discrepancies between the time in which a MRSA nasal PCR assay was ordered and the time the swab was collected, with some patients being excluded from the study due to the time between the collection date of the MRSA nasal swab and the date of the clinical culture exceeding 3 days, but the order date of the nasal swab falling within the 3-day window. This factor also poses the question of how many patients were potentially missed by restricting the inclusion criteria to ±3 days. Other strategies published in the literature include intervals of 7 or even 30 days.^4,5^ Lastly, we acknowledge that challenges may exist from the perspective of infection prevention programs depending on an institution’s policies regarding isolation of patient with MRSA colonization or infection.

In conclusion, the results of this study suggest that a negative MRSA nasal swab obtained within 3 days of a culture has a high NPV for MRSA infections in blood, bone, and soft tissues. These data support efforts to systematically evaluate the role of MRSA nasal PCR assays to help guide the appropriateness of antibiotic de-escalation beyond pneumonia.
